# Acid sphingomyelinase mediates human CD4^+^ T-cell signaling: potential roles in T-cell responses and diseases

**DOI:** 10.1038/cddis.2017.360

**Published:** 2017-07-27

**Authors:** Aiping Bai, Yuan Guo

**Affiliations:** 1 Department of Gastroenterology, The First Affiliated Hospital, Nanchang University, Nanchang 330006, Jiangxi, China

## Abstract

Acid sphingomyelinase (ASM) is a lipid hydrolase. By generating ceramide, ASM had been reported to have an important role in regulating immune cell functions inclusive of macrophages, NK cells, and CD8^+^ T cells, whereas the role of ASM bioactivity in regulation of human CD4^+^ T-cell functions remained uncertain. Recent studies have provided novel findings in this field. Upon stimulation of CD3 and/or CD28, ASM-dependent ceramide signaling mediates intracellular downstream signal cascades of CD3 and CD28, and regulates CD4^+^ T-cell activation and proliferation. Meanwhile, CD39 and CD161 have direct interactions with ASM, which mediates downstream signals inclusive of STAT3 and mTOR and thus defines human Th17 cells. Intriguingly, ASM mediates Th1 responses, but negatively regulates Treg functions. In this review, we summarized the pivotal roles of ASM in regulation of human CD4^+^ T-cell activation and responses. ASM/sphingolipid signaling may be a novel target for the therapy of human autoimmune diseases.

## Facts

- ASM is a membrane lipid hydrolase, functions to generate ceramide by hydrolyzing sphingomyelin and mediate cell signals.
- As a plasma membrane protein, ASM interacts with a variety of proteins or/and receptors.
- ASM mediates CD3 and CD28 signals, and determines T-cell activation and proliferation.
- ASM regulates pathogenic Th1 and Th17 differentiation and responses, whereas negates Treg functions.
- Blockade of ASM activities abrogates aberrant immune responses, and exhibits a novel target for the therapy of human autoimmune diseases.

## Open Questions

- Beyond our current findings, which other membrane proteins and signals does ASM interact with and mediate through?
- What the pivotal roles of ASM in mediating T-cell responses in human diseases?
- Can ASM/ceramide signals become the promising targets for treatment of human immune diseases?

Acid sphingomyelinase (ASM) belongs to the lipid hydrolase family and acts to degrade sphingomyelin to ceramide.^1^ ASM localizes to cell membranes and lysosomes, and has physical interactions with a variety of transmembrane proteins.^2, 3, 4, 5^ As the substrate of ASM, sphingomyelin is one of the main plasma membrane components, particularly the outer membrane leaflet,^1^ and provides substantial source of ceramide. It is postulated that, upon extracellular stimulations by extracellular stimuli, transmembrane proteins exhibit structural changes, resulting in relocation of themselves and other interacted proteins, i.e., through the interactions with those proteins, ASM translocates to the outer membrane leaflet.^6, 7^ Subsequently, ASM bioactivity becomes activated to catalyze hydrolysis of sphingomyelin and generate ceramide.^1^ Ceramide is an important and active lipid messenger, which mediates a variety of intracellular signals.^8, 9^ Once generated, ceramide will accumulate at plasma membrane to form ceramide-enriched membrane microcluster, leading to initiation of downstream signals.^10^ Through the generation of ceramide, ASM has an important role in regulating cell differentiation, proliferation, and apoptosis.^1, 11^

As indicated by its name, ASM has been supposed to function optimally at the cellular acidified environments, which are seen in lysosomes or under anaerobic conditions.^1^ Additional studies have shown that sphingomyelin within low-density lipoprotein (LDL) particles can be hydrolyzed to ceramide by secretory form of ASM at pH 7.4.^12^ The data indicate the broad functional conditions of ASM, either the acidified microenvironments or the physiological status.

ASM controls cellular levels of sphingomyelin and ceramide, and determines cell functions. Abnormalities in ASM bioactivity result in disturbed sphingomyelin degradation and ceramide generation, leading to extreme accumulation of sphingomyelin but deficient ceramide production.^1, 13^ Aberrant ASM-dominated sphingomyelin-ceramide signaling is associated with numbers of human nervous disorders including Alzheimer disease, Parkinson disease, schizophrenia,and depression.^13^ In particular, ASM deficiency contributes to lipid storage disorders, i.e., Niemann–Pick disease.^11, 14^

## ASM Determines Immune Cell Functions

ASM has the key role in maintaining immune homeostasis. Patients with Niemann–Pick disease, who have mutations in the ASM gene, exhibit neurological symptoms and/or visceral organ abnormalities.^11^ Meanwhile, Niemann–Pick disease patients have been reported to be susceptible to pathogen infections,^15^ indicating association between ASM deficiency and aberrant immune responses. In parallel, ASM-deficient mice display the altered levels of sphingomyelin and ceramide in the tissues and suffer exacerbated infection,^16, 17, 18^ suggesting the potential recession of immune responses associated with ASM deficiency. Further studies show this phenotype has been attributed to phagocyte dysfunction.^19^

Recently, ASM bioactivity in regulation of innate immune cell functionalities, phagocyte in particular, has been explored. ASM bioactivity in macrophage is responsible for induction or/and augmentation of inflammatory signals and cytokine production, which are likely triggered by bacterial components, e.g., lipopolysaccharide (LPS),^20, 21, 22^ saturated fatty acid such as palmitic acid,^20^ or oxidized lipids including LDL.^23^ Consequently, ASM activity determines macrophage functionalities and participates in inflammatory responses of immune diseases. Inhibition of ASM bioactivity suppresses inflammatory cytokine production from macrophages and protects animals against diseases, e.g., experimental colitis, pulmonary inflammation, and sepsis.^22, 24, 25^

ASM is also linked with other immune cell functions including those of NK cells and CD8^+^ T cells. In NK cells, plasma membrane protein CD161 interacts directly with ASM.^2^ Stimulation of NK cells by the crosslinked anti-CD161 antibodies activates ASM enzymatic activity, induces ceramide generation and subsequent intracellular signals inclusive of protein kinase B (Akt), and thus regulates NK cell functions.^2^ ASM may also mediate interleukin (IL)-2 deprivation-induced apoptosis signaling in NK cells, via generation of lysosomal ceramide.^26^

Intriguingly, ASM has been reported to regulate cytotoxic activity of CD8^+^ T cells through regulation of cytotoxic granule secretion and cytotoxic effector molecules expulsion.^27^ In ASM-knockout CD8^+^ T cells, contraction of cytotoxic granules fused to the plasma membrane is dampened, resulting in reduced release of cytotoxic effector molecules, and impaired exocytosis of cytotoxic granules and cytotoxicity.^27^ Meanwhile, ASM-deficient CD8^+^ T cells exhibit decrease of cytokine production including interferon (IFN)*γ*,^27^ indicating the pivotal role of ASM in regulation of CD8^+^ T-cell activation and functions. Our further studies show ASM controls NADH oxidase-dependent generation of reactive oxygen species and signaling in CD8^+^ T cells.^28^

## ASM Mediates CD4^+^ T-Cell Signaling

In comparison with other immune cells, ASM bioactivity in modulation of human CD4^+^ T-cell function remains largely undetermined. It is noted that by hydrolyzing sphingomyelin and generating ceramide, ASM serves as a regulator of intracellular signaling in human CD4^+^ T cells.^29, 30^ However, the exact mechanism by which pathways ASM participates in T-cell receptor (TCR)/CD3 or/and CD28 signaling remains controversial.^31, 32, 33^

Stoffel *et al.*^33^ reported that TCR and CD28 signals were independent of ASM/ceramide. In their studies, anti-CD3/CD28 antibodies, concanavalin A, and the superantigen staphylococcal enterotoxin B had been used to stimulate murine wildtype and ASM-deficient CD4^+^ T cells respectively. Upon those stimulations, ASM-deficient CD4^+^ T cells exhibited lower IL-2 secretion, but increase of intracellular IL-2 levels,^33^ suggestive of ceramide generation by ASM potentially involved in perturbation of IL-2 secretory system, other than in IL-2 production. Given IL-2 as one of the markers relevant to T-cell signaling and activation,^34^ in response to stimulations by anti-CD3 and anti-CD28 antibodies, ASM-deficient splenocytes produced comparable amount of IL-2 in comparison with wildtype cells, indicating that CD3 and CD28 signals were independent of ASM bioactivity and ceramide generation.^33^ In addition, Stoffel *et al.*^33^ further observed the reduced proliferation of ASM null CD4^+^ T cells after treatments of those stimulations, suggesting undetermined roles of ASM in CD4^+^ T cells.

In contrast, Tischner *et al.*^35^ have recently reported that ASM is requisite for protection of effector T cells against glucocorticoid-induced cell death. Glucocorticoid can induce a variety of T cells undergoing apoptosis, whereas TCR stimulation and engagement triggers survival signals and rescues the cells from death.^36^ CD4^+^ T cells in ASM-deficient mice are vulnerable to glucocorticoid-induced cell death in comparison with those wildtype cells, which mechanisms are likely associated with decreased IL-2 secretion.^35^ As IL-2 is an important cytokine related to T-cell function and activation,^34^ particularly in response to TCR stimulation, putative involvement of ASM in mediating T-cell signaling and function is implicated.

The data from the studies on ASM-deficient mice vary, and are likely suboptimal, i.e., the compensatory changes of other sphingomyelinase subtypes including neutral sphingomyelinase in the ASM null mice to be determined, as we noted the association between neutral sphingomyelinase and CD3/CD28 molecules (data not shown).

By determination of ASM bioactivities, researchers have associated ASM/ceramide production with TCR/CD28 signaling. Boucher *et al.*^32^ have noted engagement of ASM in CD28 signaling. CD28 engagement by its antibody induces significant ASM activation and ceramide generation in human T cells.^32^ Another group repeated the studies with exogenous addition of ASM or ceramide analog. Treatment with ceramide analog C6-ceramide imitated the CD28 signaling in T cells, and induced T-cell proliferation and IL-2 expression,^37^ indicating the putative roles of ASM in mediating TCR/CD28 signaling. However, the molecular mechanisms as to how ASM regulates human CD4^+^ T-cell functions are still largely unexplored.

Recently, we have reported our novel findings that ASM regulates human CD4^+^ T-cell activation and drives cell functions. ASM interacts with the intracellular domains of both CD3 and CD28, respectively.^4^ Upon stimulation of antibodies against CD3 and/or CD28, by generating ceramide, ASM mediates intracellular downstream signaling components of both CD3 and CD28, inclusive of CD3-ZAP70-PLC-*γ*1-MAPK/JNK^38, 39^ and CD28-PI3K-Akt-mTOR.^40, 41^ Consequently, human CD4^+^ T-cell activation and proliferation induced by anti-CD3/28 antibodies are characterized by increments of ASM bioactivity and ceramide production, indicating the pivotal role of ASM/ceramide in mediating CD3/CD28 intracellular signaling. In contrast, inhibition of ASM activity by pharmacological inhibitors or knockdown using small hairpin RNA (shRNA) abrogates CD3/CD28 signal cascades, and diminishes CD4^+^ T-cell activation and proliferation. Our studies indicate the potential for association of plasma membrane ASM with T-cell proteins CD3 and CD28, which are responsible for CD4^+^ T-cell activation and proliferation.

Beyond direct interaction with membrane CD3/CD28 molecules, ASM has been reported to mediate numerous signals upon certain extracellular stimulations. Proinflammatory cytokines, i.e, tumor necrosis factor (TNF), are capable to negate TCR signals, and impair T-cell activation and functions, via its type 1 receptor (TNFR1).^42^ Exposure to TNF leads to increase of ASM activity but inhibition of Ca^2+^ influx and responses in Jurkat cells in a time-dependent manner, which effects can be mimicked by exogenous supplement of ASM or ceramide.^31^ In addition, suppression of Ca^2+^ responses by TNF/TNFR1 is absent in ASM-deficient murine T cells.^31^ The study indicates that ASM involves in TNF-induced inhibition of TCR and Ca^2+^ signals in T cells.

ASM has also been reported to mediate CD95 signals. Kirschnek *et al.*^43^ have observed that, in response to anti-CD95 antibody, hepatocytes or splenocytes in wildtype mice undergo apoptosis, whereas those cells in ASM-knockout mice exhibit resistance to apoptosis. The data indicate the potential of ASM in mediating CD95-induced apoptosis-related signaling, whereas some questions still remain, i.e., any interactions between CD95 and ASM molecules. Meanwhile, determination of T-cell activation in ASM-deficient mice is another interesting question.

As one of plasma membrane proteins, ASM mediates numerous cell signals initiated by a variety of stimuli. Upon stimulations, ASM hydrolyzes sphingomyelin and generates ceramide. Subsequently, ceramides redistribute and accumulate at plasma membrane rapidly to form ceramide-enriched domains and initiate downstream signals.^10^ Given the pivotal roles of ASM activity and ceramide redistribution in changing membrane domains and mediating cell signals,^7^ we propose broad interactions of ASM with numbers of membrane proteins and receptors. Consequently, through the interactions with those membrane proteins, ASM/ceramide signaling activates relevant signals in T cells and exhibits distinct functions in responses to certain extracellular stimuli.

## ASM Dominates Th1 Response

T helper cells (Th) have the pivotal roles in adaptive immune responses. Once activated by anti-CD3/28 antibodies or major histocompatibility complex (MHC) stimulation, CD4^+^ T cells differentiate into specific Th cell subsets including Th1, Th2, Th17, and regulatory T cells (Treg) under an appropriate cytokine milieu, i.e., IL-12, IL4, IL-6 or/and transforming growth factor-*β* (TGF*β*).^44, 45^ Our subsequent studies have demonstrated that human CD4^+^ T-cell differentiation, i.e., Th1 is characterized by increments in ASM bioactivity and ceramide production, and phosphorylation of proteins including c-Jun N-terminal kinases (JNK) and Akt, whereas inhibition of ASM activity by its inhibitors or shRNA knockdown blocks those cell signals and abrogates Th1 cell differentiation.^4^

In addition, regulation of murine Th1 differentiation by ceramide has also been reported. Ceramide analog, i.e. C6-ceramide, has been noted to enhance IL-12-induced Th1 differentiation.^46^ C6-ceramide increases T-bet and cyclooxygenase (COX)2 expressions in murine CD4^+^ T cells, and sequently augments IFN*γ* production, whereas COX2 specific inhibitor overrides C6-ceramide-induced Th1 generation in a dose-dependent manner,^46^ indicating COX2 likely as another downstream pathway of ASM/ceramide regulating Th1 differentiation. These data provide evidence that, by generating ceramide, ASM activity not only mediates human and murine CD4^+^ T-cell activation but also regulates T-cell differentiation and Th1 responses.

## ASM Determines Human Th17 Response

Among Th cells, Th17 cells have been recently established as a unique cell lineage, which are critical for persistent immune responses and contribute to the pathogenesis of chronic inflammatory disorders.^30, 47^ A variety of intracellular signals inclusive of mammalian target of rapamycin (mTOR) and signal transducer and activator of transcription 3 (STAT3) are key putative elements associated with Th17 cell differentiation, and determine Th17 cell functions.^48, 49^

We have recently noted that dual expression of CD39 and CD161 defines human IL-17 producing Th17 cells.^3^ Intriguingly, CD39 is coexpressed with CD161 in human CD4^+^ T cells, and both plasma membrane proteins associate directly with ASM. Stimulation by anti-CD39 or/and CD161 antibodies augments ASM activity, induces cellular ceramide production, and mediates downstream signaling cascades including STAT3 and mTOR,^3^ which are indispensible and integral elements for Th17 generation.^48, 49^ Mechanistically, in the presence of Th17 differentiation cytokines such as IL-6, MHC/CD3/28 stimulation induces ASM activities, which mediate mTOR and STAT3 signaling in CD4^+^ T cells. Stimulation of CD39 and CD161 further amplifies ASM-mediated mTOR and STAT3 signals, which eventually drive Th17 generation.^30^ These findings suggest a novel cellular link between transmembrane proteins such as CD39/CD161 and ASM-dependent ceramide signals in driving Th17 expansion in autoimmune diseases.

**Figure 1:**
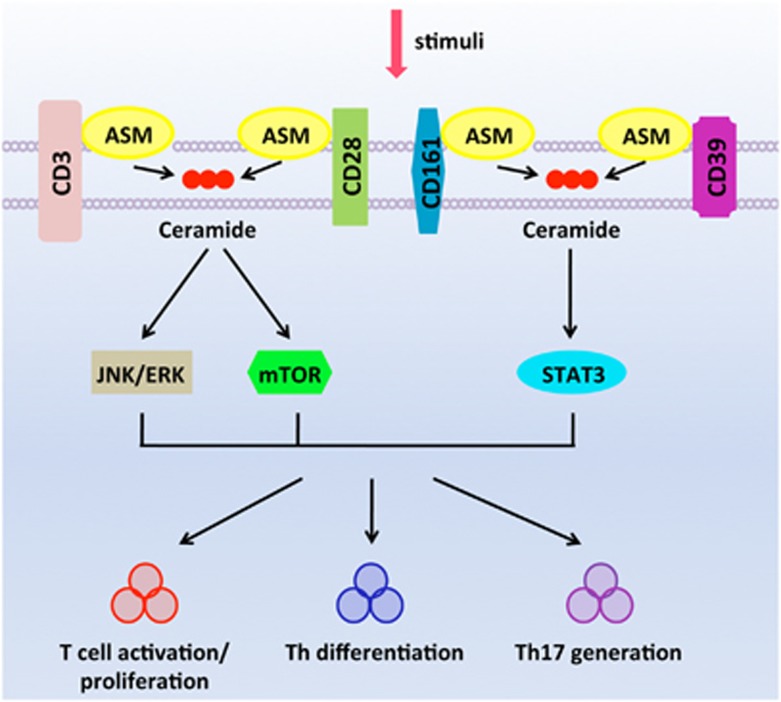
ASM mediates CD4^+^ T-cell signaling and determines Th responses. ASM has physical interactions with cell surface receptors including CD3, CD28, CD39, and CD161. Stimulations of those surface receptors, i.e., CD3 and CD28, amplify ASM bioactivity, and activate downstream signals inclusive of JNK/ERK and mTOR. Meanwhile, upon ligations of CD39 and CD161, ASM/recemide signals induce activation of mTOR and STAT3. Consequently, those activated signals exhibit synergistic effects to drive T-cell activation and induce Th responses

Th17 cells have been implicated in the pathogenesis of human immune diseases, e.g., Crohn’s disease.^50^ We have further noted that inhibitors of ASM block both STAT3 and mTOR signals and limit Th17 responses in the inflamed tissues of patients with active Crohn’s disease.^3^ The data indicate the pivotal role of ASM-dependent intracellular signaling transduction in mediating T-cell activation and promoting Th17 responses in human diseases, as illustrated in Figure 1.

## ASM Regulates Treg Functions

Treg cells are a group of CD4^+^ T-cell subsets with suppressive activities, which act to regulate immune cell functions, maintain or/and induce tolerance to antigens, and inhibit immune responses in human diseases.^51^ Forkhead box P3 (FOXP3), a transcriptional factor of the forkhead box superfamily, is a master signal protein in regulation of Treg differentiation, development, and functions.^52^ It is noted that intermediate TCR stimulation triggered by adequate amount of antigens can induce FOXP3 expression, through a variety of intracellular signal pathways including nuclear factor-*κ*B (NF*κ*B) and forkhead box O (Foxo).^53^ Conversely, phosphoinositide 3-kinase (PI3K)-Akt signaling in CD4+ T cells, likely initiated by CD28 ligation, dampens FOXP3 expression and diminishes Treg differentiation.^54, 55^ Given the pivotal role of ASM in mediating TCR and CD28 intracellular signals, regulation of Treg functions by ASM is proposed.

A recent study has noted ASM as a negative regulator of Treg development.^56^ In comparison with low levels of Treg cells in wildtype mice, ASM-deficient mice have higher numbers of systemic Treg cells. In parallel, ASM deficiency in CD4^+^ T cells correlates with impaired PI3K-Akt signal activation, resulting in augmentation of FOXP3 expression as well as induction of Treg differentiation.^56^ The data suggest that decrease or deficiency of ASM activities in CD4^+^ T cells is associated with Treg expansion. Another study has further confirmed that frequency of Treg and their suppressive activities associated CTLA4 are increased in ASM null mice.^57^ Intriguingly, ASM activity and ceramide contents are elevated in Treg cells in contrast to other CD4^+^ T effector cells. ASM activities, likely associated with CD28 signaling, negatively correlate with suppressive functions of Treg.^57^ Blockade of ASM activities by its inhibitors and supplement of IL-2 induce FOXP3 expression and Treg numbers *in vivo*.^57^ The data above indicate the putative roles of ASM in negating Treg functions.

## Potential Roles of ASM in Immune Diseases

Immune diseases are a group of human immune disorders including diabetes, Crohn’s disease, lupus, psoriasis, and myocarditis. Those diseases display abnormal immune responses, some with excessive autoimmunity against tissue antigens which are physically present in tissues or/and organs of human body, as seen in autoimmune diseases.^58^ It is supposed that under certain circumstance such as breakdown of anergy system, self antigens are exposed to and recognized by immune cells, followed by activation of immune cells and initiation of immune responses.^59, 60^ The etiology of autoimmune diseases is still unknown. However, some infections in the early life of the patients may present bacterial antigens to immune system, which share the similar antigenic domain or sequences with healthy tissue proteins.^61^ Consequently, those tissue proteins may induce self-reactive immune responses, self-reactive and pathogenic T cells in particular, through molecular mimicry or sequestration mechanisms.^61, 62^

Autoimmune diseases are characterized by excessive immune responses. Aberrant CD4^+^ T-cell responses have been implicated in the development of autoimmune diseases, and also associated with disease courses.^63, 64^ Therapeutic interventions targeting pathogenic T cells have been introduced clinically for the treatment of autoimmune diseases, with promising clinical efficacy.^65^ Besides its role in regulation of innate immune cell functions, ASM activity is associated with T-cell signals and Th responses, and may involve in the pathogenesis of autoimmune diseases.

ASM has interactions with multiple cell membrane proteins including CD3 and CD28, as shown in Table 1. Through those interactions, ASM mediates intracellular signals which determinate T-cell activation and pathogenic Th cell differentiation, which are inclusive of Th1, Th2, and Th17.^65^ In addition, ASM can also negatively regulate Treg functions,^56^ resulting in breakdown of immune tolerance. Given the potential role of ASM in regulation of T-cell responses, blockade of ASM/ceramide signaling may become an alternative target for the management of autoimmune diseases.

A recent study has associated cellular ceramide production with murine T-cell activity and graft-*versus*-host disease (GVHD) development. Ceramide generation by another synthases, i.e., ceramide synthase 6, is requisite for TCR signal transduction including ZAP70 activation in T cells, as well as T-cell activation and proliferation *in vivo*.^66^ Meanwhile, during GVHD development, elevated ceramide production augments pathogenic Th1 responses and increases proinflammatory cytokine levels including IFN*γ*.^66^ Conversely, inhibition of ceramide generation diminishes T-cell responses *in vivo* and *in vitro*, and attenuates disease activity of GVHD model.^66^

As discussed above and summarized in Table 2, ASM activity and ceramide production are associated with T-cell activation and pathogenic Th cell generation, and thus contribute to aberrant autoimmune responses. ASM/ceramide signals appear as the promising treatment targets for inflammation and autoimmune diseases. However, clinical employment of ASM/ceramide inhibition strategies is likely beyond what we proposed. For instance, clinical pilot study has shown that inhibition of ASM or ASM deficiency does not protect from GVHD in transplant recipients with Niemann–Pick disease.^67^ Meanwhile, considering the broad signals mediated by ASM/ceramide in response to diverse stimuli, inhibition of ASM may impact a variety of cell functions, some indispensable for maintenance of physical conditions of human body. In this manner, blockade of ASM/ceramide signals may induce some unexpected adverse effects. Thus, to avoid any potential side effects, pinpointing ASM activities in distinct cells would optimize pharmacological development of ASM inhibition for clinical use.

## Conclusions

**Figure 2:**
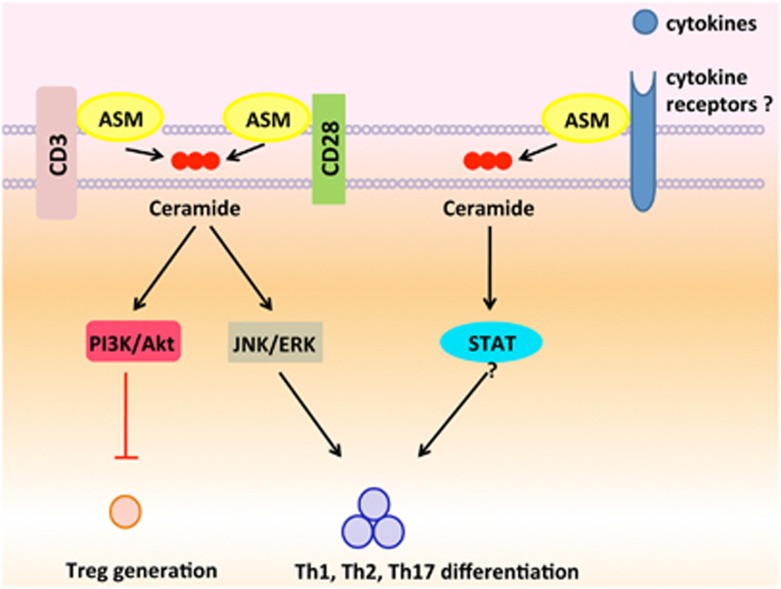
ASM determinates Th responses. Upon stimulations of TCR and CD28 by MHC or antibody ligation, ASM is activated to mediate T-cell activation, whereas PI3K-Akt signaling, likely downstream of CD28 pathways, dampens Treg generation. Meanwhile, a variety of membrane receptors inclusive of CD161, CD39, and perhaps some cytokine receptors, interact with ASM. Once those membrane receptors ligated by their ligands/cytokines, ASM/ceramide signals become activated, and initiate the key pathways which are indispensible for T-cell differentiation and pathogenic Th responses

So far, a variety of cellular membrane proteins/receptors have been reported to interact with ASM, including CD3, CD28, CD39, and CD161.^2, 3, 4, 32^ Given its multiple bio-functions, i.e., mediating TNF receptor or CD95 signals,^31, 43^ interactions of ASM with those receptors (possibly beyond those receptors reported) are likely proposed. Upon stimulations of cellular receptors inclusive of TCR/CD3 and CD28, ASM is activated, and thus mediates numerous downstream signals, which are responsible for T-cell activation. Meanwhile, ligations of other receptors inclusive of CD161, CD39, and perhaps some cytokine receptors, by their ligands or cytokines, activate ASM/ceramide signals, and initiate the key pathways, which are indispensible for pathogenic T-cell responses (Figure 2). As T-cell responses have pivotal roles in human immune diseases, inhibition of ASM bioactivity blocks CD4^+^ T-cell signaling, and dampens pathogenic Th responses. ASM activities and signaling may provide potential alternatives to current therapeutic approaches to control CD4^+^ T-cell responses, and may help to design new strategies for treatment of human autoimmune diseases.

## Publisher's Note

Springer Nature remains neutral with regard to jurisdictional claims in published maps and institutional affiliations.

## Figures and Tables

**Table 1:** Potential membrane proteins interact with ASM on T cells

**Membrane proteins**	**Downstream signals**	**Effects of ASM on the signals**	**References**
CD3	ZAP70-PLC-*γ*1-MAPK/JNK	T-cell activation, proliferation, differentiation; IL-2 production	4
CD28	PI3K-Akt-mTOR	T-cell activation, proliferation, differentiation; IL-2 production	4, 32
TNFR1?	Ca^2+^ signal	Inhibition of Ca^2+^ influx and responses	31
CD95?	Apoptosis-related signal	Mediating CD95-induced apoptosis	43
CD39, CD161	mTOR, STAT3	Induction of human Th17	3

**Table 2:** Models targeting ASM in T cells

**Model name**	**Effects of targeting ASM**	**References**
ASM-knockout CD8^+^T cells	Impaired exocytosis of cytotoxic granules, diminished cytotoxicity, decreased IFN*γ* production	27
ASM-deficient CD4^+^ T cells	Perturbated IL-2 secretion Reduced CD4^+^ T-cell proliferation	33
ASM-deficient mice	Decreased IL-2 production by CD4^+^ T cells Increase of glucocorticoid-induced cell death	35
ASM-deficient mice	Reduced CD95-induced apoptosis of splenocytes	43
ASM-deficient mice	Impaired PI3K-Akt signal Augmentation of Treg function	56
Human graft-*versus*-host disease	No protect of ASM deficiency in transplant recipients with Niemann–Pick disease	67
